# Disease and Treatment-Related Burden in Patients With Acromegaly Who Are Biochemically Controlled on Injectable Somatostatin Receptor Ligands

**DOI:** 10.3389/fendo.2021.627711

**Published:** 2021-03-15

**Authors:** Maria Fleseriu, Mark Molitch, Alexander Dreval, Nienke R. Biermasz, Murray B. Gordon, Ross D. Crosby, William H. Ludlam, Asi Haviv, Yossi Gilgun-Sherki, Susan D. Mathias

**Affiliations:** ^1^ Pituitary Center, Departments of Medicine and Neurological Surgery, Oregon Health and Science University, Portland, OR, United States; ^2^ Division of Endocrinology, Metabolism and Molecular Medicine, Northwestern University Feinberg School of Medicine, Chicago, IL, United States; ^3^ Department of Clinical Endocrinology, Vladimirsky Moscow Regional Research Clinical Institute, Moscow, Russia; ^4^ Department of Medicine, Division of Endocrinology, Leiden University Medical Center, Leiden, Netherlands; ^5^ Allegheny Neuroendocrinology Center, Allegheny General Hospital, Pittsburgh, PA, United States; ^6^ Outcomes Research, Health Outcomes Solutions, Winter Park, FL, United States; ^7^ Biomedical Statistics, Sanford Center for Biobehavioral Research, Fargo, ND, United States; ^8^ Clinical Development, Chiasma, Inc., Needham, MA, United States

**Keywords:** acromegaly, quality of life, patient-reported outcome, Acro-TSQ, somatostatin receptor ligands, burden

## Abstract

Medical treatment for acromegaly commonly involves receiving intramuscular or deep subcutaneous injections of somatostatin receptor ligands (SRLs) in most patients. In addition to side effects of treatment, acromegaly patients often still experience disease symptoms even when therapy is successful in controlling GH and IGF-1 levels. Symptoms and side effects can negatively impact patients’ health-related quality of life. In this study, we examine the disease- and treatment-related burden associated with SRL injections as reported through the use of the Acromegaly Treatment Satisfaction Questionnaire (Acro-TSQ ^©^) and clinician-reported symptom severity through the Acromegaly Index of Severity (AIS). Patients included in this analysis were enrolled in a randomized phase 3 study, were biochemically-controlled (an IGF-1 < 1.3 × the upper limit of normal [ULN] and average GH < 2.5 ng/ml) and receiving SRL injections for ≥6 months with a stable dose of either long-acting octreotide or lanreotide monotherapy for ≥4 months. The sample (N = 91) was 65% female, 91% Caucasian, with a mean [standard deviation (SD)] age of 53 (1) years. Two-thirds of patients reported that they still experience acromegaly symptoms; 82% of these said they experience symptoms all of the time. Three-fourths experienced gastrointestinal (GI) side effects after injections, and 77% experienced treatment-related injection site reactions (ISRs). Patients commonly reported that these interfered with their daily life, leisure, and work activities. Those with higher symptom severity, as measured by the AIS, scored significantly worse on several Acro-TSQ domains: Symptom Interference, GI Interference, Treatment Satisfaction, and Emotional Reaction. Despite being biochemically controlled with injectable SRLs, most patients reported experiencing acromegaly symptoms that interfere with daily life, leisure, and work. GI side effects and ISRs were also common. This study highlights the significant disease burden that still persists for patients with acromegaly that have achieved biochemical control with the use of injectable SRLs.

## Introduction

Acromegaly is a rare hormonal disorder most often caused by a benign tumor of the pituitary gland (2–4). Excess production of growth hormone (GH) and insulin-like growth factor 1 (IGF-1) can result in symptoms such as changes in appearance and enlargement of hands and feet, as well as fatigue, joint pain, and systemic complications including cardiovascular disease and respiratory disease (5–7). Somatostatin receptor ligands (SRLs) are the most common first-line medical treatment (8–11) and are administered as either long-acting intramuscular (octreotide LAR) or deep subcutaneous injections (lanreotide autogel). SRLs are successful in achieving biochemical control in just over half of those treated (1). Among patients partially responsive to SRLs, higher doses, more frequent administration, or combination therapy with dopamine agonists or GH receptor antagonists, may improve biochemical control (12–15). Common side effects of treatment can include injection site reactions (ISRs) and gastrointestinal (GI) symptoms (16, 17). Despite achieving biomedical control, as defined by IGF-1 and GH levels, acromegaly symptoms may persist in many patients (18). These symptoms, in addition to treatment-related side effects, can negatively impact patients’ health-related quality of life (HRQoL) (18–21).

Previous studies have examined these impacts using patient reported outcomes (PROs) and observed significant burden from both acromegaly and its treatment (7, 18, 22). In addition to evaluating biochemical control, PROs have been identified as key components for monitoring disease and assessing treatment effectiveness (23).

The current study analyzes data from the Screening phase of a global, randomized phase 3 study assessing oral *versus* injectable SRLs in acromegaly patients (MPOWERED) (24). In addition to assessing the patient burden associated with SRL injections, incorporating PRO measures into a clinical trial setting provides the opportunity to examine PROs alongside clinical measures of disease severity. The current analysis examines the disease- and treatment-related burden associated with SRL injections as reported through the use of the Acromegaly Treatment Satisfaction Questionnaire (Acro-TSQ) (24), and clinician-reported symptom severity through the Acromegaly Index of Severity (AIS).

## Materials and Methods

### Study Type and Patient Population

Patients were enrolled in a randomized phase 3 MPOWERED (Maintenance of Acromegaly Patients with Octreotide Capsules Compared With Injection-Evaluation of REsponse Durability) study (NCT02685709); results presented herein reflect data collected during the Screening phase only of this study. Biochemically controlled acromegaly patients receiving SRL injections for ≥6 months and a stable dose for ≥4 months of either long-acting octreotide or lanreotide monotherapy were eligible. Biochemical control was defined as having an IGF-1 assay <1.3 times the age-adjusted upper limit of normal (ULN) and an average of five GH values <2.5 ng/ml.

Patients were recruited from numerous countries; those represented in these analyses include Austria (7%), France (8%), Lithuania (3%), the Russian Federation (56%), Serbia (4%), Spain (6%), and the United States (15%).

### Measurements

Patients completed the Acro-TSQ questionnaire (25), a PRO measure developed specifically for use with acromegaly patients receiving oral or injectable treatment. Its questions assess several domains: Symptom Interference, GI Side Effect Interference, Injection Site Interference, Treatment Satisfaction, Emotional Reaction, and Treatment Convenience. It has previously been found to be reliable and valid; minimally important differences (MIDs)—which represent the smallest changes that patients would deem important—have been estimated for some of the domains (26–28).

The AIS reflects symptoms routinely assessed in clinical trials (29–31). This tool, which reflects the clinician’s assessment of symptoms based on what the patient describes, was used in this analysis to examine clinician-reported symptoms of acromegaly. The AIS contains five items assessing the following symptoms: headache, swelling of extremities, joint pain, sweating, and fatigue. Each symptom is graded by its highest severity during the last four weeks, from no symptoms (score 0), to mild symptoms (2), moderate symptoms (3) or severe symptoms (4). The AIS Overall Score is the sum of severity scores for each of the five symptoms, with scores ranging from 0 (no symptoms) to 15 (severe symptoms).

### Statistical Analysis

Demographic and clinical characteristics including disease duration, injectable SRL treatment, IGF-1 and GH levels, the number of active symptoms, and AIS scores were summarized through descriptive analysis. Frequencies of each response option for the Acro-TSQ were examined. These item-level data address the presence and frequency of symptoms, the type and timing of side effects, emotional reactions to treatment, and overall satisfaction with current treatment. Acro-TSQ domain scores were computed and compared across subgroups based on age, gender, time since diagnosis, AIS score, IGF-1 and GH levels, and prior treatment (SRL injection type and dose). Two age cutoffs were used; 55 (the median of the sample) and 35 (to identify a younger subgroup) years of age. Differences in domain scores were evaluated based on statistical significance (p < 0.05) as well as by comparison with available MID estimates. Whether the existence of GI side effects differed by baseline characteristics was also explored.

## Results

### Patient Characteristics

146 patients were enrolled in the Screening phase. The final version of the Acro-TSQ was not available at the start of the study; 91 (62%) completed the final version of the Acro-TSQ and were therefore eligible for the current analysis. The sample was 65% female, and 91% Caucasian. The mean (SD) age was 53 (1) years, and the mean (SD) time since diagnosis was 11 (9) years. At screening, 65% of patients had an IGF-1 ≤1 × ULN, with a mean (SD) IGF-1 level of 0.86 (0.26). Approximately three-fourths of patients (76%) had >3 active symptoms on the AIS, with mean (SD) total AIS scores of 4.97 (3.21) of a possible maximum of 15 (**Table 1**). For purposes of this analysis, those with an AIS score <5 were considered to have low severity and those with scores ≥5 were considered to have moderate to high severity.

**Table 1:** Patient characteristics.

Characteristic	
Total, N	91
Age, years (mean, SD)	53 (11)
Female Gender (n, %)	59 (64%)
Time since acromegaly diagnosis, years mean (SD)	11 (8)
Injectable SRL Treatment n (%) (n=89)	
Lanreotide (60 mg)	8 (9%)
Lanreotide (90 mg)	7 (8%)
Lanreotide (120 mg)	16 (18%)
Octreotide (10 mg)	5 (6%)
Octreotide (20 mg)	30 (33%)
Octreotide (30 mg)	23 (25%)
IGF-1 ULN (mean, SD)	0.86 (0.26)
GH ng/ml (mean, SD)	0.80 (0.62)
Active Symptoms at Screening (n, %)	
1	3 (3%)
2	3 (3%)
3	16 (18%)
>3	69 (76%)
AIS (mean, SD)	4.97 (3.2) Range: 0–13

AIS, Acromegaly index of severity; SD, standard deviation; SRL, Somatostatin receptor ligands.

### Item-Level Results

Thirty-six patients (40%) responded that their current injectable SRL treatment improves symptoms, but 61 (67%) indicated that they still experience acromegaly symptoms. Of these 61, 82% said they experience symptoms all the time, 48% said symptoms emerge or worsen before their next dose, and 95% indicated that they were bothered by the amount of time they experienced symptoms. Patients frequently reported that acromegaly symptoms interfered with daily life (56/61 = 92%), leisure activities (51/61 = 84%), and work activities (46/53 = 87%) (**Table 2**).

**Table 2:** Impact of symptoms and GI side effects.

Acro-TSQ Item	Not at all	A little	Somewhat	Quite a bit	Very much
Symptoms interfere with daily activities (n = 61)	8% (n = 5)	31% (n = 19)	38% (n = 23)	19% (n = 12)	3% (n = 2)
Symptoms interfere with leisure activities (n = 61)	16% (n = 10)	34% (n = 21)	30% (n = 18)	11% (n = 7)	8% (n = 5)
Symptoms interfere with work activities* (n = 53)	13% (n = 7)	34% (n = 18)	32% (n = 17)	15% (n = 8)	6% (n = 3)
GI side effects interfere with daily activities (n = 66)	35% (n = 23)	32% (n = 21)	20% (n = 13)	12% (n = 8)	2% (n = 1)
GI side effects interfere with leisure activities (n = 67)	33% (n = 22)	37% (n = 25)	18% (n = 12)	10% (n = 7)	2% (n = 1)
GI side effects interfere with work activities** (n = 60)	43% (n = 23)	36% (n = 19)	17% (n = 9)	15% (n = 8)	2% (n = 1)
Bothered by ISR during the first few days (n = 70)	33% (n = 23)	50% (n = 35)	9% (n = 6)	6% (n = 4)	3% (n = 2)
ISR interfered with daily life (n = 70)	43% (n = 30)	31% (n = 22)	23% (n = 16)	1% (n = 1)	1% (n = 1)
Bothered by scheduling injections (n = 88)	45% (n = 40)	33% (n = 29)	10% (n = 9)	8% (n = 7)	3% (n = 3)
Bothered by traveling for injections (n = 82)	24% (n = 20)	27% (n = 22)	26% (n = 21)	15% (n = 12)	9% (n = 7)

*Excludes five patients who do not work for pay because of acromegaly and five patients who do not work for pay because of other reasons.

**Excludes three patients who do not work for pay because of acromegaly and three patients who do not work for pay because of other reasons.

GI, Gastrointestinal; ISR, Injection site reaction.

Approximately three-fourths of patients (74%) reported experiencing GI side effects after injections. The length of time after an injection these side effects were experienced ranged from 0 to 56 days, with a mean (SD) of 8 (9.7) days (data not shown). GI side effects interfered with daily life for 65% (43/66), leisure activities for 67% (45/67), and work activities for 62% (37/60) of patients (**Table 2**).

Seventy patients (77%) experienced treatment-related ISRs (*e.g.*, pain, swelling, itching, bruising, *etc*.). Of these patients, 67% (47/70) were bothered by ISRs during the first few days, and 57% (40/70) said they interfered with daily life (**Table 2**).

The proportions of patients who felt sad or anxious about getting treatment were 53 and 51%, respectively; 47% reported being frustrated about how they received their treatment, and 64% were upset about being dependent on others (data not shown). Among all patients, 55 and 76% were bothered by having to schedule and travel for injections, respectively (**Table 2**).

More than half of patients reported that they were either satisfied (41.8%) or very satisfied (15.4%) with their current treatment overall; 26.4% said they were somewhat satisfied, 6.6% were neither satisfied nor dissatisfied, and the remainder (9.9%) were somewhat to very dissatisfied. The average (SD) rating for overall satisfaction was 2.59 (1.28) (1 = very satisfied, 4 = neither satisfied nor dissatisfied, 7 = very dissatisfied), which is approximately midway between “Somewhat Satisfied” and “Satisfied” (data not shown).

### Domain Scores

The individual domains of the Acro-TSQ allow for a closer examination of specific aspects of acromegaly and its treatment. When comparing Acro-TSQ domain scores by patient demographics and clinical characteristics, no significant differences were observed by the analyzed subgroups of gender, age, disease duration, or medication dose. However, the difference in the domain score of Symptom Interference by gender (10.4) suggests a trend toward statistical significance (p = 0.059), with women reporting more Symptom Interference (68.9) than men (79.3) (lower scores indicate higher burden, **Table 3**).

**Table 3:** Acro-TSQ domains by patient characteristics.

Comparison	Symptom Interference*mean (SD)	Treatment Convenience*mean (SD)	Injection Site Interference*mean (SD)	GI Interference*mean (SD)	Treatment Satisfaction*mean (SD)	Emotional Reaction*mean (SD)
Female	68.9 (24.8) N = 59	66.4 (22.1) N = 59	81.8 (20.7) N = 59	79.1 (23.8) N = 59	62.7 (20.0) N = 59	79.5 (21.2) N = 59
Male	79.3 (25.2) N = 32	69.0 (17.1) N = 32	83.6 (23.6) N = 32	82.0 (24.0) N = 32	62.9 (18.5) N = 32	77.1 (25.2) N = 32
p-value	0.059	0.561	0.705	0.577	0.950	0.626
Age ≤ 55	70.4 (25.1) N = 45	66.7 (21.0) N = 45	84.2 (19.8) N = 45	79.4 (26.0) N = 45	64.6 (16.1) N = 45	76.5 (24.4) N = 45
Age > 55	74.6 (25.5) N = 46	67.9 (20.0) N = 46	80.7 (23.4) N = 46	80.8 (21.7) N = 46	61.0 (22.2) N = 46	80.8 (20.7) N = 46
p-value	0.434	0.769	0.449	0.788	0.382	0.366
Age ≤ 35	74.3 (17.0) N = 9	58.8 (20.7) N = 9	73.6 (22.9) N = 9	71.3 (24.0) N = 9	64.5 (17.3) N = 9	70.4 (19.1) N = 9
Age > 35	72.3 (26.1) N = 82	68.2 (20.3) N = 82	83.4 (21.4) N = 82	81.1 (23.7) N = 82	62.6 (19.7) N = 82	79.6 (22.9) N = 82
p-value	0.825	0.189	0.200	0.243	0.778	0.249
Disease Duration < 5 years	72.2 (23.9) N = 22	65.3 (25.1) N = 22	82.4 (26.6) N = 22	81.1 (25.1) N = 22	61.5 (18.3) N = 22	75.4 (23.2) N = 22
Disease Duration ≥ 5 years	72.6 (25.9) N = 69	67.9 (18.8) N = 69	82.4 (20.0) N = 69	79.8 (23.5) N = 69	63.2 (19.9) N = 69	79.7 (23.2) N = 69
p-value	0.938	0.606	0.994	0.834	0.727	0.437
AIS < 5	81.8 (23.7) N = 43	70.3 (20.1) N = 43	86.3 (20.2) N = 43	88.2 (16.7) N = 43	69.2 (15.0) N = 43	83.7 (18.4) N = 43
AIS ≥ 5	64.2 (23.9) N = 48	64.7 (20.5) N = 48	78.9 (22.5) N = 48	72.9 (26.9) N = 48	57.0 (21.2) N = 48	74.1 (25.2) N = 48
p-value	**0.001**	0.194	0.102	**0.002**	**0.002**	**0.045**
Octreotide	70.2 (25.1) N = 58	65.4 (19.1) N = 58	83.0 (21.4) N = 58	81.6 (21.6) N = 58	57.5 (18.6) N = 58	81.3 (20.0) N = 58
Lanreotide	75.2 (25.5) N = 31	71.8 (22.5) N = 31	82.7 (22.3) N = 31	76.3 (27.9) N = 31	72.0 (17.6) N = 31	75.0 (26.7) N = 31
p-value	0.371	0.161	0.949	0.326	**0.001**	0.211
IGF-1 ≤ 1xULN	76.3 (24.1) N = 59	67.9 (19.5) N = 59	80.5 (23.4) N = 59	80.8 (23.6) N = 59	65.8 (17.3) N = 59	79.4 (21.7) N = 59
IGF-1 > 1xULN	65.6 (26.4) N = 32	66.3 (22.3) N = 32	85.9 (17.9) N = 32	78.9 (24.5) N = 32	57.1 (22.0) N = 32	77.3 (24.4) N = 32
p-value	0.055	0.725	0.256	0.720	**0.040**	0.684
IGF-1 ≤ 1xULN and mean GH < 1 ng/ml	79.5 (24.9) N = 42	70.0 (19.7) N = 42	80.1 (23.6) N = 42	83.1 (24.7) N = 42	68.3 (16.7) N = 42	79.0 (23.7) N = 42
IGF-1 >1xULN or mean GH ≥ 1 ng/ml	66.6 (24.3) N = 49	65.0 (20.9) N = 49	84.4 (19.9) N = 49	77.6 (23.0) N = 49	58.0 (20.4) N = 49	78.4 (21.8) N = 49
p-value	**0.015**	0.239	0.339	0.267	**0.011**	0.906
Lanreotide dose:**						
Low	76.6 (29.3) N = 8	62.5 (28.4) N = 8	85.9 (12.4) N = 8	71.9 (35.1) N = 8	66.7 (20.4) N = 8	56.3 (33.9) N = 8
Middle	62.5 (22.5) N = 7	70.2 (23.9) N = 7	87.5 (12.5) N = 7	63.1 (29.2) N = 7	63.9 (16.0) N = 7	75.0 (23.1) N = 7
High	80.1 (24.5) N = 16	77.1 (18.3) N = 16	78.9 (28.8) N = 16	84.4 (21.9) N = 16	78.1 (15.4) N = 16	84.4 (19.9) N = 16
p-value	0.321	0.331	0.635	0.216	0.124	**0.046**
Octreotide dose:**						
Low	80.0 (24.0) N = 5	70.0 (13.6) N = 5	77.5 (22.4) N = 5	85.0 (14.9) N = 5	58.9 (9.5) N = 5	91.7 (8.3) N = 5
Middle	68.8 (25.4) N = 30	64.7 (21.1) N = 30	81.3 (21.5) N = 30	81.7 (24.0) N = 30	59.4 (14.9) N = 30	83.3 (17.1) N = 30
High	69.8 (25.5) N = 23	65.2 (17.8) N = 23	86.4 (21.6) N = 23	80.8 (20.0) N = 23	54.6 (24.0) N = 23	76.4 (24.2) N = 23
p-value	0.656	0.852	0.581	0.927	0.641	0.226

*Lower scores indicate greater interference/lower satisfaction; higher scores indicate less interference/higher satisfaction.

**“Low” is any octreotide dose <20 mg total/month or lanreotide <90 mg total/month; “Middle” is any octreotide dose <30 mg total/month or lanreotide <120 mg total/month; “High” is any octreotide dose ≥30 mg total/month or lanreotide ≥120 mg total/month.

AIS, Acromegaly index of severity; GI, Gastrointestinal; SD, standard deviation; ULN upper limit of normal. Bold values represent statistical significance (p < 0.05).

Treatment Satisfaction was significantly better for those with both an IGF-1 level ≤1 × ULN and a GH level < 1 ng/ml *versus* those with either IGF-1 >1 × ULN or GH ≥1 ng/ml. When stratified by IGF-1 ≤1 × ULN *vs >*1 × ULN, the difference in Symptom Interference approached statistical significance (p = 0.055). When the criteria were expanded to having an IGF-1 level >1 × ULN or a GH level ≥1 ng/ml, Symptom Interference was significantly worse than for those with both an IGF-1 level ≤1 × ULN and GH level <1 ng/ml. In the latter case, the observed difference in Symptom Interference (12.9) exceeded the estimated MID value (10–12 points) (26).

To examine whether domain scores differed by clinician-reported disease severity, patients were stratified into two AIS categories (low = 0–4 and moderate to high = ≥5). Symptom Interference, GI Interference, Treatment Satisfaction, and Emotional Reaction were significantly worse for those with AIS scores ≥5. Additionally, the differences for Symptom Interference (17.6) and GI Interference (15.3) exceeded MID estimates (10–12 points and 8–10 points, respectively (26) for these two domains (**Table 3**). The difference in Treatment Satisfaction was 6.4, which did not exceed the previously estimated MID of 9–11 points.

Scores for Treatment Satisfaction were significantly higher (better) for long-acting lanreotide users versus long-acting octreotide users, and for those with an IGF-1 level ≤ 1xULN versus >1xULN.

### Analyses of GI Side Effects

Upon closer examination of GI side effects, they were found to be more common among those who experienced acromegaly symptoms. Specifically, among patients experiencing acromegaly symptoms, 75% also experienced GI side effects, compared to 67% of those who were not experiencing acromegaly symptoms (not significant, data not shown). Analyses were conducted to determine whether demographic and clinical characteristics differed between those experiencing (N = 66) *versus* not experiencing (N = 25) GI side effects. No significant differences were observed between groups at screening in terms of gender, age, ethnicity, duration of illness, IGF-1 ULN, GH levels, or total AIS scores (data not shown).

## Discussion

The Acro-TSQ, which assesses disease and treatment burden as well as treatment satisfaction, has been previously shown to be valid and reliable (26) for patients with acromegaly, and its scales correlate with other well-known measures (the Treatment Satisfaction Questionnaire for Medication [TSQM] (32), AcroQoL (33), AIS, Work Productivity and Activity Impairment Questionnaire Specific Health Problem V2.0 [WPAI:SHP] (34), and the 5-level EQ-5D version [EQ-5D-5L] (35).

Notably, in this study, most patients treated with injectable SRLs and considered well controlled (IGF-1 ≤1.3 × ULN) experienced acromegaly symptoms despite being biochemically controlled on medical treatment. Additionally, most patients reported that symptoms interfered with daily life and negatively impacted leisure and work activities. GI side effects and ISRs were also common, lasting an average of 8 days (and as many as 56 days) after an injection, and interfered with daily life. Over half the patients reported that they feel sad (53%) and anxious (51%) about getting their treatment; the majority were bothered by having to schedule and travel to receive their injections. Interestingly, despite this, patients are relatively satisfied with their treatment overall and the mode of administration and associated ISRs do not appear to be the main driver of satisfaction.

Symptom Interference, GI Interference, Treatment Satisfaction, and Emotional Reaction domain scores were significantly worse for those with AIS scores ≥5 *versus* those with AIS scores <5. Additionally, the observed differences in Symptom Interference and GI Interference for those with AIS ≥5 *vs <*5 exceeded MID estimates, demonstrating that these differences are clinically meaningful. This suggests that there is a relationship between several Acro-TSQ domains and patient- and clinician-perceived acromegaly severity as measured by AIS. The relationship between GI Interference and AIS, in particular, was unexpected and suggests that GI Interference may be influenced by both treatment-related and disease-related symptoms. We also showed that Symptom Interference was significantly worse for those with either IGF-1 >1 × ULN or GH ≥1 ng/ml *versus* those with both IGF-1 ≤1 × ULN and GH <1 ng/ml, indicating that patients with greater biochemical response reported experiencing less impact from their symptoms. Interestingly, Treatment Satisfaction was significantly higher for long-acting lanreotide *versus* long-acting octreotide users, probably related to mode of administration/self-administration; no other domains differed significantly by type of SRL treatment.

Other studies of acromegaly patients receiving injectable SRL treatment have also reported symptom persistence and side effects associated with treatment. In a study by Strasburger et al. (18), 70% of patients reported acromegaly symptoms despite treatment. While satisfaction was not reported by type of treatment, patients reported longer pain duration and higher incidence of nodules after long-acting octreotide injections than for long-acting lanreotide injections. In addition to their SRL treatment, in that study 21.1% were also receiving dopamine agonists, and 16.6% received pegvisomant. However, only 36.4% of patients were biochemically controlled (defined as having an age-normalized IGF-1 standard deviation score ≤2.0), compared with 65% in our current study. In a study by Salvatori et al. (36), 59 patients receiving long-acting lanreotide injections reported a variety of GI side effects, including diarrhea (44%), abdominal pain (14%), nausea (14%), and flatulence (7%); several ISRs were reported including pain (10%), irritation (8%), pruritus (7%). IGF-1 control was obtained in 72.4% of patients by the end of the study; 59% had glucose-suppressed GH levels <1 µg/L, though utility of oral glucose tolerance testing in patients on SRLs has been questioned (37). In a randomized, double-blind study of pasireotide and long-acting octreotide, the frequency of GI side effects for octreotide patients were: diarrhea (45%), abdominal pain (22%), nausea (22%), and abdominal distension (12%), which were all more common than in pasireotide patients (30). Control rates in that study at month 12 for both IGF-1 and GH (<2.5 µg/L) were 38.6 and 48.3% for pasireotide patients and 23.6 and 51.6% for long-acting octreotide patients, respectively. The PAOLA trial, a large international study of patients uncontrolled initially on long term octreotide or lanreotide, reported data on both short- and long-term GI side effects of injectable SRLs. In the initial 24-week trial, patients using long-acting octreotide or lanreotide experienced diarrhea (8%), abdominal pain (3%), and nausea (3%) (38). For those receiving pasireotide, those percentages were 18% (diarrhea), 8% (abdominal pain), and nausea (5%). More recently, results from an extension of this trial offer insight into long-term use of pasireotide (up to 5.8 years). Among 187 patients (including those who were switched to pasireotide from either long-acting octreotide or lanreotide after the initial 24-week trial), 22% experienced diarrhea, 14% abdominal pain, 9% nausea, and 5% vomiting (39).

In a US-based study, 105 acromegaly patients treated with injectable SRLs in routine clinical practice reported experiencing a variety of symptoms; 80% reported four or more symptoms. The most common were acro-fog (defined by the authors as “a short-term memory loss or feeling in a daze”) (83%), joint pain (83%), soft tissue swelling (81%), fatigue/weakness/tiredness (80%), and headaches (75%). Several symptoms were reported as occurring “constantly” by a majority of patients. The most common ISRs included pain during the injection (83%), pain for several hours after the injection (68%), and nodules at the injection site (63%) (7). Another survey assessed HRQoL among 106 US acromegaly patients and revealed that only 56% of those receiving injections for acromegaly were satisfied with their treatment (19). Furthermore, long-term use of SRLs has previously been shown to be associated with worse physical functioning, fatigue, and HRQoL (21).

Although our study included data from a large sample of patients from seven different countries around the world and patients completed questionnaires without knowing their final laboratory values and/or if they had qualified for enrollment into the Run-in phase of the study, a few limitations should be noted. To begin, there is the potential for selection bias because only patients who had an interest in switching away from their current treatment would likely enroll in the study. Further, only patients who met biochemical criteria (IGF-1 <1.3 × ULN and GH <2.5 ng/ml) at screening and receiving SRL injections for ≥6 months with a stable dose for ≥4 months were included.

Despite being on a stable dose of long-acting SRLs, patients report acromegaly symptoms that interfere with their daily life, leisure and work activities. We also showed that Symptom Interference was significantly lower in patients when both IGF-1 level ≤1 × ULN and GH level ≤1 compared with patients with elevated levels for either IGF-1 or GH, highlighting the importance of achieving complete biochemical control. GI side effects were also common, and patients report that GI side effects interfere with daily activities, including leisure and work. Some patient-reported measures of symptom and treatment burden were more severe among those with higher clinician-reported symptom severity (AIS) and clinical indications of lower biochemical response, as measured by IGF-1 and GH. This study highlights the significant disease burden that still persists for patients with acromegaly in a large international population that have achieved biochemical control with the use of injectable SRLs.

## Data Availability Statement

The raw data supporting the conclusions of this article will be made available by the authors, without undue reservation.

## Ethics Statement

The studies involving human participants were reviewed and approved by local and central IRBs. The patients/participants provided their written informed consent to participate in this study.

## Author Contributions

WL and AH conceived the study. WL, AH, MF, RC, and SM were responsible for the design of the study. RC conducted the statistical analysis. MF, MM, and SM wrote and corrected the manuscript. All authors contributed to the article and approved the submitted version.

## Funding

This study was funded by Chiasma, Inc.

## Conflict of Interest

MF has received research support from Chiasma, Crinetics, Ionis, Novartis, and Recordati and is a scientific consultant to Chiasma, Crinetics, Ionis, Ipsen, Novartis, Pfizer, and Recordati. MG has received research support from Chiasma, Corcerpt, Camurus, Crinetics, Ipsen, Novartis, OPKO, Pfizer, Strongbridge, Teva, and Novo Nordisk and is a scientific consultant to Chiasma and Novo Nordisk. MM has received research support from Chiasma, Crinetics, Ionis, and Novartis, and is a consultant to Chiasma, Pfizer, and Merck. WL, AH, and YG-S are employed by and own stock in Chiasma, Inc. SM is an employee of Health Outcomes Solutions (HOS), which received funding from Chiasma, Inc. for conducting these analyses. RC is a consultant to HOS, which received funding from Chiasma, Inc. for conducting these analyses.

The remaining authors declare that the research was conducted in the absence of any commercial or financial relationships that could be construed as a potential conflict of interest.

## References

[1] Carmichael JD Bonert VS Nuño M Ly D Melmed S. Acromegaly clinical trial methodology impact on reported biochemical efficacy rates of somatostatin receptor ligand treatments: a meta-analysis. J Clin Endocrinol Metab (2014) 99(5):1825–33. 10.1210/jc.2013-3757 PMC4010703 24606084

[2] Ben-Shlomo A Melmed S. Acromegaly. Endocrinol Metab Clinics North America (2008) 37(1):101–22. 10.1016/j.ecl.2007.10.002 PMC2697616 18226732

[3] Melmed S. Acromegaly pathogenesis and treatment. J Clin Invest (2009) 119(11):3189–202. 10.1172/JCI39375 PMC2769196 19884662

[4] Melmed S Colao A Barkan A Molitch M Grossman AB Kleinberg D. Guidelines for acromegaly management: an update. J Clin Endocrinol Metab (2009) 94(5):1509–17. 10.1210/jc.2008-2421 19208732

[5] Gadelha MR Kasuki L Lim DST Fleseriu M. Systemic Complications of Acromegaly and the Impact of the Current Treatment Landscape: An Update. Endocrine Rev (2019) 40(1):268–332. 10.1210/er.2018-00115 30184064

[6] Biermasz NR. The burden of disease for pituitary patients. Best Pract Res Clin Endocrinol Metab (2019) 33(2):101309. 10.1016/j.beem.2019.101309 31405752

[7] Geer EB Sisco J Adelman DT Ludlam WH Haviv A Liu S. Patient reported outcome data from acromegaly patients treated with injectable somatostatin receptor ligands (SRLs) in routine clinical practice. BMC Endocrine Disord (2020) 20(117). 10.1186/s12902-020-00595-4 PMC7393879 32736547

[8] Galoiu S Poiana C. Current therapies and mortality in acromegaly. J Med Life (2015) 8(4):411–5. 10.1371/journal.pone.0036411 PMC4656943 26664461

[9] Melmed S Bronstein MD Chanson P Klibanski A Casanueva FF Wass JAH. A Consensus Statement on acromegaly therapeutic outcomes. Nat Rev Endocrinol (2018) 14(9):552–61. 10.1038/s41574-018-0058-5 PMC7136157 30050156

[10] Giustina A Barkan A Beckers A Biermasz N Biller BMK Boguszewski C. A Consensus on the Diagnosis and Treatment of Acromegaly Comorbidities: An Update. J Clin Endocrinol Metab (2019) 105(4). 10.1210/jc.2012-1833 31606735

[11] Giustina A Barkhoudarian G Beckers A Ben-Shlomo A Biermasz N Biller B. Multidisciplinary management of acromegaly: A consensus. Rev Endocrine Metab Disord (2020) 21(4):667–78. 10.1007/s11154-020-09588-z PMC7942783 32914330

[12] Giustina A Bonadonna S Bugari G Colao A Cozzi R Cannavo S. High-dose intramuscular octreotide in patients with acromegaly inadequately controlled on conventional somatostatin analogue therapy: a randomised controlled trial. Eur J Endocrinol (2009) 161(2):331–8. 10.1530/EJE-09-0372 19465485

[13] Giustina A Mazziotti G Cannavò S Castello R Arnaldi G Bugari G. High-Dose and High-Frequency Lanreotide Autogel in Acromegaly: A Randomized, Multicenter Study. J Clin Endocrinol Metab (2017) 102(7):2454–64. 10.1210/jc.2017-00142 28419317

[14] Bonert V Mirocha J Carmichael J Yuen KCJ Araki T Melmed S. Cost-Effectiveness and Efficacy of a Novel Combination Regimen in Acromegaly: A Prospective, Randomized Trial. J Clin Endocrinol Metab (2020) 105(9). 10.1210/clinem/dgaa444 32754748

[15] Fleseriu M Biller BMK Freda PU Gadelha MR Giustina A Katznelson L. A Pituitary Society update to acromegaly management guidelines. Pituitary (2020) 24:1–13. 10.1007/s11102-020-01091-7 33079318 PMC7864830

[16] Sandostatin (octreotide acetate) injection [package insert]. East Hanover, NJ: Novartis Pharmaceuticals Corporation (1998).

[17] Somatuline depot (lanreotide) injection [package insert]. Ipsen Biopharmaceuticals: BAsking Ridge, NJ (2007).

[18] Strasburger CJ Karavitaki N Stormann S Trainer PJ Kreitschmann-Andermahr I Droste M. Patient-reported outcomes of parenteral somatostatin analogue injections in 195 patients with acromegaly. Eur J Endocrinol (2016) 174(3):355–62. 10.1530/EJE-15-1042 PMC4722610 26744896

[19] Liu S Adelman DT Xu Y Sisco J Begelman SM Webb SM. Patient-centered assessment on disease burden, quality of life, and treatment satisfaction associated with acromegaly. J Invest Med: Off Publ Am Fed Clin Res (2018) 66(3):653–60. 10.1136/jim-2017-000570 29151042

[20] Kepicoglu H Hatipoglu E Bulut I Darici E Hizli N Kadioglu P. Impact of treatment satisfaction on quality of life of patients with acromegaly. Pituitary (2014) 17(6):557–63. 10.1007/s11102-013-0544-7 24337714

[21] Postma MR Netea-Maier RT van den Berg G Homan J Sluiter WJ Wagenmakers MA. Quality of life is impaired in association with the need for prolonged postoperative therapy by somatostatin analogs in patients with acromegaly. Eur J Endocrinol (2012) 166(4):585–92. 10.1530/EJE-11-0853 22250074

[22] Geer EB Sisco J Adelman DT Ludlam WH Haviv A Gelbaum D. Observed discordance between outcomes reported by acromegaly patients and their treating endocrinology medical provider. Pituitary (2020) 23(2):140–8. 10.1007/s11102-019-01013-2 PMC7066283 31808101

[23] Langlois F Suarez GM Fleseriu M. Updates in rare and not-so-rare complications of acromegaly: focus on respiratory function and quality of life in acromegaly. F1000Research (2020) 9. 10.12688/f1000research.22683.1 PMC7391012 32765836

[24] Fleseriu M Katznelson L Clemmons D Trainer P Biermasz N Strasburger C eds. MPOWERED: Study design of a phase 3 head-to-head trial evaluating oral octreotide capsules versus injectable somatostatin analogs in patients with acromegaly. In: American Association of Clinical Endocrinologists (AACE) 25th Annual Scientific and Clinical Congress. Orlando, FL. p. 25–9.

[25] Fleseriu M Fogelfeld L Gordon MB Sisco J Colwell HH Ludlam WH. Development of a novel patient-reported measure for acromegaly: the Acro-TSQ. Pituitary (2019) 22(6):581–93. 10.1007/s11102-019-00986-4 PMC6842345 31522359

[26] Fleseriu M Fogelfeld L Gordon MB Sisco J Crosby RD Ludlam WH. An evaluation of the Acromegaly Treatment Satisfaction Questionnaire (Acro-TSQ) in adult patients with acromegaly, including correlations with other patient-reported outcome measures: data from two large multicenter international studies. Pituitary (2020) 23(4):347–58. 10.1007/s11102-020-01038-y PMC7316852 32221764

[27] Fleseriu M Fogelfeld L Gordon NB Sisco J Ludlam WH Mathias SD. Evaluation of Acromegaly Symptoms from the newly developed AcromegalyTreatment Satisfaction Questionnaire (Acro-TSQ): a prospective patient-reported outcome study. In: 99th Annual Meeting of the Endocrine Society (ENDO). Orlando, FL (2017).

[28] Fleseriu M Fogelfeld L Gordon NB Sisco J Colwell HH Haviv A. The ACRO-TSQ - A Novel Patient-Reported Tool to Assess Satisfaction with Acromegaly Treatment. In: The 8th International Congress of the GRS and IGF Society. Tel Aviv, Isreal.

[29] Caron P Beckers A Cullen DR Goth MI Gutt B Laurberg P. Efficacy of the new long-acting formulation of lanreotide (lanreotide Autogel) in the management of acromegaly. J Clin Endocrinol Metab (2002) 87(1):99–104. 10.1210/jcem.87.1.8153 11788630

[30] Colao A Bronstein MD Freda P Gu F Shen CC Gadelha M. Pasireotide versus octreotide in acromegaly: a head-to-head superiority study. J Clin Endocrinol Metab (2014) 99(3):791–9. 10.1210/jc.2013-2480 PMC3965714 24423324

[31] Petersenn S Farrall AJ De Block C Melmed S Schopohl J Caron P. Long-term efficacy and safety of subcutaneous pasireotide in acromegaly: results from an open-ended, multicenter, Phase II extension study. Pituitary (2014) 17(2):132–40. 10.1007/s11102-013-0478-0 PMC3942632 23529827

[32] Atkinson MJ Sinha A Hass SL Colman SS Kumar RN Brod M. Validation of a general measure of treatment satisfaction, the Treatment Satisfaction Questionnaire for Medication (TSQM), using a national panel study of chronic disease. Health Qual Life Outcomes (2004) 2:12. 10.1186/1477-7525-2-12 14987333 PMC398419

[33] Badia X Webb SM Prieto L Lara N. Acromegaly Quality of Life Questionnaire (AcroQoL). Health Qual Life Outcomes (2004) 2:13. 10.1186/1477-7525-2-13 14987332 PMC404471

[34] Reilly MC Zbrozek AS Dukes EM. The validity and reproducibility of a work productivity and activity impairment instrument. PharmacoEconomics (1993) 4(5):353–65. 10.2165/00019053-199304050-00006 10146874

[35] Herdman M Gudex C Lloyd A Janssen M Kind P Parkin D. Development and preliminary testing of the new five-level version of EQ-5D (EQ-5D-5L). Qual Life Res An Int J Qual Life Aspects Treatment Care Rehab (2011) 20(10):1727–36. 10.1007/s11136-011-9903-x PMC3220807 21479777

[36] Salvatori R Nachtigall LB Cook DM Bonert V Molitch ME Blethen S. Effectiveness of self- or partner-administration of an extended-release aqueous-gel formulation of lanreotide in lanreotide-naive patients with acromegaly. Pituitary (2010) 13(2):115–22. 10.1007/s11102-009-0207-x PMC2855862 19898989

[37] Carmichael JD Bonert VS Mirocha JM Melmed S. The utility of oral glucose tolerance testing for diagnosis and assessment of treatment outcomes in 166 patients with acromegaly. J Clin Endocrinol Metab (2009) 94(2):523–7. 10.1210/jc.2008-1371 19033371

[38] Gadelha MR Bronstein MD Brue T Coculescu M Fleseriu M Guitelman M. Pasireotide versus continued treatment with octreotide or lanreotide in patients with inadequately controlled acromegaly (PAOLA): a randomised, phase 3 trial. Lancet Diabetes Endocrinol (2014) 2(11):875–84. 10.1016/S2213-8587(14)70169-X 25260838

[39] Colao A Bronstein MD Brue T De Marinis L Fleseriu M Guitelman M. Pasireotide for acromegaly: long-term outcomes from an extension to the Phase III PAOLA study. Eur J Endocrinol (2020) 182(6):583. 10.1530/EJE-19-0762 32217809 PMC7222286

